# Peripheral absolute eosinophil count identifies the risk of serious immune-related adverse events in non-small cell lung cancer

**DOI:** 10.3389/fonc.2022.1004663

**Published:** 2022-10-13

**Authors:** Yan Wu, Dapeng Li, Mengyao Wu, Ying Yang, Meng Shen, Kai Chen

**Affiliations:** Department of Oncology, The First Affiliated Hospital of Soochow University, Suzhou, China

**Keywords:** non-small cell lung cancer, immune-related adverse events, high-grade, predictor, prognosis, real-world evidence

## Abstract

**Background:**

Immune-related adverse events (irAEs) have drawn a lot of attention lately as a result of the predominance of immunotherapy in advanced non-small cell lung cancer (NSCLC). However, the clinical evidence for irAEs in real life is limited. In this paper, the occurrence of irAEs in Chinese NSCLC patients was examined, and possible risk factors for the emergence of severe irAEs were discovered.

**Methods:**

Our retrospective investigation assessed the occurrence of adverse events (AEs) and prognosis of 213 patients who received immunotherapy for NSCLC. Using univariate and multivariate logistic regression models, the association between clinicopathological traits and the incidence of severe irAEs was investigated. To assess the prognostic impact of irAEs, survival data was analyzed.

**Results:**

Among the 213 NSCLC patients, 122 (57.3%) had irAEs of any grade, and 38 (17.8%) had high-grade (grade 3-5) AEs. Baseline peripheral absolute eosinophil count (AEC) (HR 6.58, 95% CI: 1.5-28.8, P=0.012) was found to be an independent predictor of high-grade irAEs by multivariate analysis. The survival analysis revealed that patients with severe irAEs had worse OS (15.7 vs. 20.8 months, 95% CI: 11.6-19.8 vs. 16.0-25.5, P=0.026).

**Conclusion:**

According to our findings, the peripheral absolute eosinophil count (AEC) is a reliable indicator of severe irAEs in NSCLC. Serious irAEs that occur in patients often reflect poor prognoses. In the future, high-grade irAEs should receive more attention.

## Introduction

Non-small cell lung cancer (NSCLC) contributes to more than 80% of lung cancers, which is also a major cause of cancer-related mortality globally (1, 2). Over the past 10 years, there have been more available treatment choices for patients with NSCLC. For patients with positive driver gene mutations, targeted therapy has radically altered the treatment strategy. Chemotherapy has been the classic treatment for those without driver genes. However, recent advancements in programmed death 1/programmed death ligand 1 (PD-1/PD-L1) inhibitors have given NSCLC patients additional options (3, 4).

The effectiveness of immunotherapy in treating NSCLC has greatly increased as a result of its ability to prevent the inhibitory effects that tumors have on immune cells (5). In advanced NSCLC, two monoclonal antibodies against PD-1, pembrolizumab and nivolumab, as well as a monoclonal antibody against PD-L1, atezolizumab, have been approved as first or second-line treatments (6, 7). Recent clinical trials have shown that these drugs can significantly prolong patients’ survival time compared with conventional chemotherapy (8). However, while suppressing tumor progression, immune checkpoint inhibitors (ICIs) therapy also disrupts the immune balance of various systems in the body, causing immune-related adverse events (irAEs).

The Common Toxicity Criteria for Adverse Events (CTCAE) version 5.0 was generally used to evaluate irAEs. Most adverse events (AEs) were grade 1-2 and were usually mild and self-limiting. High-grade (grades 3-5) adverse events are often severe and even fatal (9, 10). In previous studies, baseline neutrophil-lymphocyte ratios (NLR) and platelet-lymphocyte ratios (PLR) have been found to be predictive of irAEs (11). However, until now, there have been no indicators that help predict severe irAEs.

In this paper, we investigated the occurrence and risk factors of severe irAEs in NSCLC patients using real-world data.

## Materials and methods

### Patient information

Our research comprised patients who visited the First Affiliated Hospital of Soochow University during the period January 2020-June 2021. Here are the criteria for inclusion: (1) patients whose pathology report indicates a diagnosis of NSCLC, and (2) patients who have experienced at least one cycle of immunotherapy. The following were the criteria for exclusion: (1) patients who have recently received blood-parameter-altering medication, and (2) patients whose records are incomplete. Patients’ medical records were retrieved retrospectively through the hospital’s electronic database. Age, sex, pathology type, ECOG PS, gene mutation, PD-L1 TPS, treatment regimen, lab parameters, as well as the date of death, progression, or last follow-up were noted. Laboratory parameters include baseline peripheral blood eosinophil count, neutrophil count, serum albumin content, tumor marker levels, etc. Blood is usually collected and evaluated 1 day prior to initial treatment and no more than 7 days prior to therapy. Essentially, progression-free survival (PFS) is the time from the initiation of immunotherapy until tumor progression or death. Overall survival (OS) was calculated from the start of immunotherapy to the time of death. Patients lost to follow-up or without tumor progression were considered censored. The final follow-up to ensure that patients hadn’t progressed or died was used as the censoring time. We completed the follow-up on June 1, 2022. The First Affiliated Hospital of Soochow University’s Ethics Committee granted approval for this retrospective investigation (No. 297, 2022).

### Diagnosis of irAEs

In this work, irAEs were diagnosed by two attending oncologists with more than 5 years of experience and one chief oncologist with more than 20 years of experience. According to the Technical Guidelines for Evaluation of Immune-Related Adverse Events in Antitumor Therapy issued by the Drug Review Center of the State Drug Administration of China, the criteria for adverse drug reactions that were determined to be causally related to immune mechanisms are summarized as follows: (1) whether immunosuppressive therapy or endocrine replacement therapy was used for the target irAEs (including suspected irAEs) and the regression after treatment; (2) whether the event was related to treatment in temporal correlation, including whether the event occurred after prolonged dosing and whether the event recurred or worsened after re-dosing; (3) the event was reported with the same target drug and was clearly an irAE; and (4) other plausible explanations that could lead to the target adverse event were excluded (e.g., infection, coadministration, and underlying disease). IrAEs include dermatologic, endocrinologic, pulmonary, gastrointestinal, hepatic, neurologic, hematologic, and other rare adverse events. Patients were thoroughly examined by the attending physician every 3 weeks during immunotherapy to comprehensively assess and document irAEs. IrAEs were evaluated and graded according to CTCAE version 5.0. The clinical manifestations, types, grades, date of occurrence, treatment methods, and prognosis of irAEs were retrospectively recorded.

### Statistical analysis

Using the data we gathered, we separated the research cohort into two categories: patients with irAEs and those without. With either a Fisher’s exact test or a Chi-square test, we compared the clinicopathological characteristics of the two groups. We also performed univariate and multivariate logistic regression to analyze predictors of serious irAEs. The multivariate analysis included variables with a P value of less than 0.1 in the univariate analysis. Additionally, we evaluated survival data by Kaplan-Meier and compared the differences between groups through log-rank tests. GraphPad Prism 8 and IBM SPSS 22 were used to create the graphs and analyze the data. The ‘forestploter’ package in R (v.3.6.3) was used for forest plots. All P values under 0.05 were regarded as statistically significant, which were all based on two-sided hypothesis testing.

## Results

### Patient traits

Our study covered 213 patients in total. The ages of the enrolled patients varied from 28 to 90. The patients were mostly male (85.0%). In over half (60.1%) of the patients, there was a history of smoking. The vast majority (96.7%) were clinical stage III-IV. A pathological diagnosis of NSCLC was made in all patients. Among them, the pathological types were mainly adenocarcinoma (53.1%) and squamous cell carcinoma (42.3%). ECOG PS of 0 or 1 was present in the majority (61.5%) of patients prior to therapy. In tumor tissues from over half (61.0%) of the patients, the PD-L1 gene was expressed. Only a small proportion (12.2%) of patients were found to have ALK or ECOG gene mutations. Nearly half (48.8%) of patients received immunotherapy as first-line therapy. Seventy-two (33.8%) patients received ICIs monotherapy. Anti-PD-1 immunotherapy was used in 210 (98.6%) patients. **Table 1** lists the baseline clinical features of the individuals.

**Table 1:** Characteristics of the study population.

Variables	With irAEs	Non irAEs	*P*
	(n = 122, %)	(n = 91, %)	
Age			0.348
<65	52 (42.6)	33 (36.3)	
≥65	70 (57.4)	58 (63.7)	
Gender			0.517
Female	20 (16.4)	12 (13.2)	
Male	102 (83.6)	79 (86.8)	
Smoking status			0.185
Smoker	78 (63.9)	50 (54.9)	
Never-smoker	44 (36.1)	41 (45.1)	
ECOG			0.086
0-1	69 (7.4)	62 (8.5)	
≥2	53 (92.6)	29 (91.5)	
Tumor Stage			0.838
II	5 (4.1)	2 (2.2)	
III	40 (32.8)	29 (31.9)	
IV	77 (63.1)	60 (65.9)	
Histologic type			0.749
Adenocarcinoma	64 (52.5)	49 (53.8)	
Squamous	51 (41.8)	39 (42.9)	
Others	7 (5.7)	3 (3.3)	0.995
PD-L1 expression			
<1%	14 (11.5)	10 (11)	
1-50%	32 (26.2)	24 (26.4)	
>50%	43 (35.2)	31 (34.1)	
Unknown	33 (27)	26 (28.6)	0.889
EGFR status			
Wild type	45 (36.9)	31 (34.1)	
Mutant	10 (8.2)	7 (7.7)	
Unknown	67 (54.9)	53 (58.2)	0.289
ALK status			
Wild type	99 (81.1)	66 (72.5)	
Mutant	5 (4.1)	4 (4.4)	
Unknown	18 (14.8)	21 (23.1)	
Lines of immunotherapy			0.049
First line	57 (46.7)	47 (51.6)	
Second line	53 (43.4)	27 (29.7)	
Third line and above	12 (9.8)	17 (18.7)	
Type of drug			0.262
Anti-PD-1	119 (97.5)	91 (100)	
Anti-PD-L1	3 (2.5)	0 (0)	
Therapeutic modalities			0.364
Single drug	46 (37.7)	26 (28.6)	
Combined with chemotherapy	63 (51.6)	55 (60.4)	
Others	13 (10.7)	10 (11)	

irAE, immune-related adverse events; ECOG, Eastern Cooperative Oncology Group; PD-1, programmed death 1; PD-L1, programmed death ligand 1; EGFR, epithelial growth factor receptor; ALK, anaplastic lymphoma kinase. P-values <0.05 were considered statistically significant.

### Features of irAEs

**Figure 1:**
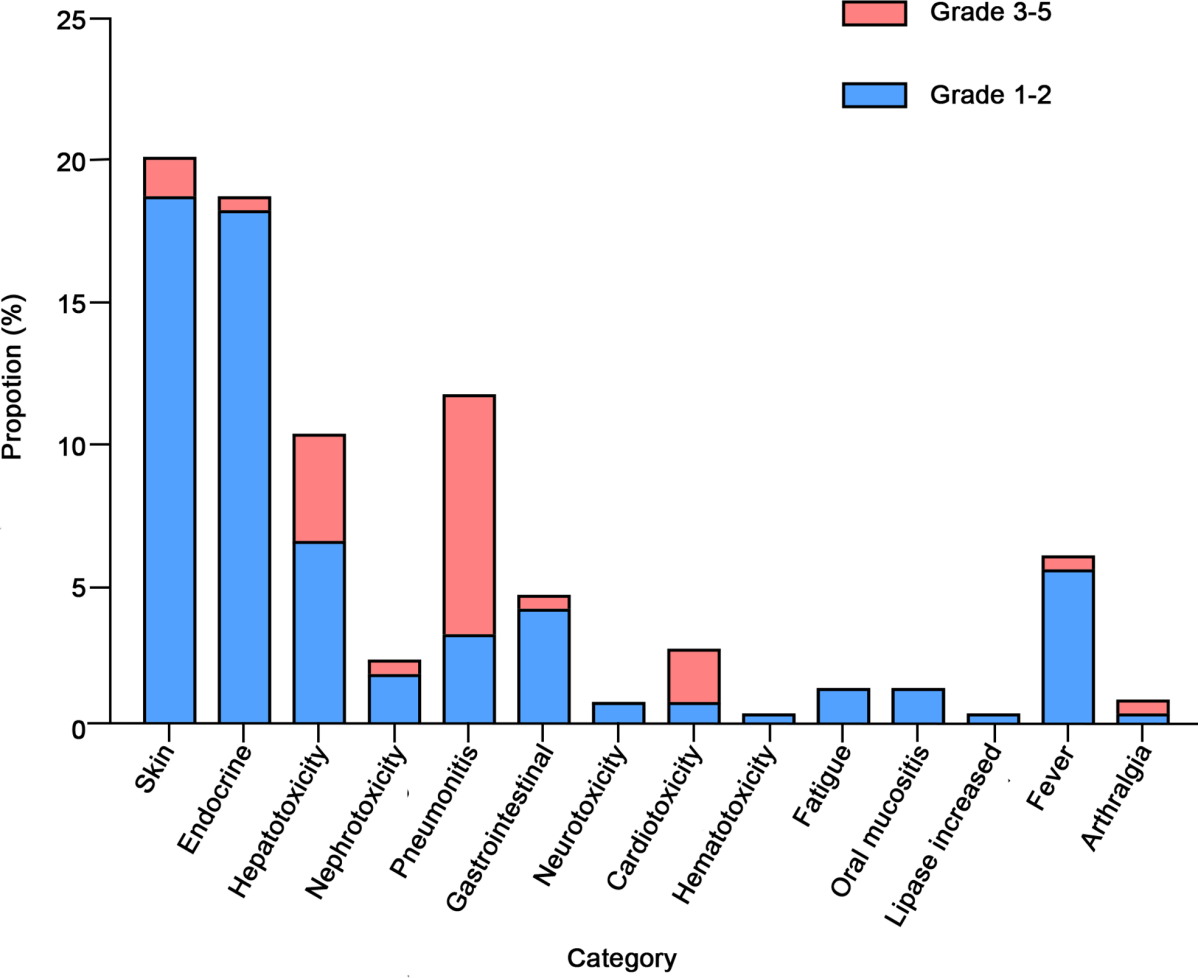
Category and grade of immune-related adverse events (irAEs) in non-small cell lung cancer (NSCLC).

As shown in **Table 2**, in our study, more than half (57.3%) experienced irAEs, and nearly one-fifth (19.7%) of patients experienced more than two types of irAEs. The most common AEs occurred in the skin (20.2%), endocrine (18.8%), lung (11.7%), and liver (10.3%) (**Figure 1**). In terms of clinical manifestations, the most common clinical manifestations were rash (14.1%), pneumonitis (11.7%), hypothyroidism (9.9%), pruritus (9.9%) and elevated transaminases (9.4%). In this cohort of 213 patients with NSCLC, nearly one-fifth (17.8%) suffered high-grade (grade 3-5) AEs. Among them, the most prevalent high-grade AEs were pneumonitis (8.5%), elevated transaminases (3.8%), cardiotoxicity (1.9%), and pruritus (1.4%).

**Table 2:** Overall adverse events.

Item	All AEs (n=213, %)	Grades 3-5 AEs (213, %)
All	122 (57.3)	38 (17.8)
Skin toxicity	43 (20.2)	3 (1.4)
Rash	30 (14.1)	2 (0.9)
Pruritus	21 (9.9)	3 (1.4)
Erythema	3 (1.4)	2 (0.9)
Desquamation	3 (1.4)	2 (0.9)
Reactive capillary hemangiomas	3 (1.4)	0
Endocrine toxicity	40 (18.8)	1 (0.5)
Hypothyroidism	21 (9.9)	1 (0.5)
Hyperthyroidism	3 (1.4)	0
Hyperglycemia	3 (1.4)	0
Hypophysitis	14 (6.6)	0
Hepatotoxicity	22 (10.3)	8 (3.8)
Transaminase increased	20 (9.4)	8 (3.8)
Blood bilirubin increased	2 (0.9)	0
Nephrotoxicity	5 (2.3)	1 (0.5)
Pneumonitis	25 (11.7)	18 (8.5)
Gastrointestinal toxicity	10 (4.7)	1 (0.5)
Diarrhea	1 (0.5)	0
Constipation	1 (0.5)	0
Nausea	4 (1.9)	0
Colitis	1 (0.5)	1 (0.5)
Poor appetite	3 (1.4)	0
Neurotoxicity	2 (0.9)	0
Cardiotoxicity	6 (2.8)	4 (1.9)
Hematotoxicity	1 (0.5)	0
Fatigue	3 (1.4)	0
Oral mucositis	3 (1.4)	0
Lipase increased	1 (0.5)	0
Fever	13 (6.1)	1 (0.5)
Arthralgia	1 (0.5)	1 (0.5)

AEs, adverse events.

### Predictors of high-grade irAEs

In our study cohort, a total of 26 patients discontinued the drug because of irAEs. Among them, up to 20 (76.9%) patients discontinued due to high-grade (grade 3-5) AEs. Therefore, we first investigated risk factors for high-grade irAEs. The univariate logistic regression revealed that ECOG score (P=0.003), peripheral absolute eosinophil count (AEC) (P=0.013), serum albumin content (P=0.044), body mass index (BMI) (P=0.025), and tumor marker CYFRA21-1 level (P=0.02) were associated with high-grade irAEs (**Table 3**). We constructed forest plots to provide a more visual depiction of the findings (**Supplementary Figures S1A, B**). As shown in the univariate forest plot, ECOG score (HR 3.02, 95%CI: 1.46-6.21, P=0.003), BMI (HR 0.87, 95% CI: 0.77-0.98, P=0.025) and AEC (HR 5.78, 95%CI: 1.45-23, P=0.013) were strongly associated with grade 3-5 irAEs. However, in the multivariate forest plot, only AEC was independently linked with grade 3-5 irAEs (HR 6.58, 95% CI 1.5-28.8, P=0.012).

**Table 3:** Univariate and multivariate analyses of factors associated with grades 3-5 irAEs.

Factors			Univariate analysis		Multivariate analysis
			OR (95%CI)		OR (95%CI)
			*P*		*P*
Age	<65 ≥65		1.55 (0.74-3.27) 0.25		
Gender	Male Female		0.93 (0.35-2.45) 0.884		
ECOG	0-1 ≥2		3.02 (1.46-6.21) 0.003		2.46 (0.89-6.81) 0.08
PD-L1 expression	<1%				
	1-50%		0.207 1 (0.33-3.02) 1		
	>50%		0.42 (0.13-1.32) 0.137		
	Unknown		0.54 (0.17-1.73) 0.3		
Lines of immunotherapy	First line		0.194		
	Second line		1.39 (0.67-2.88) 0.380		
	Third line and above		0.35 (0.08-1.62) 0.181		
Neutrophils (×10^9^/L)			1.03 (0.93-1.14) 0.588		
Eosinophils (×10^9^/L)			5.78 (1.45-23) 0.013		6.58 (1.50-28.8) 0.012
Hemoglobin (g/L)			0.99 (0.97-1.01) 0.086		0.99 (0.97-1.02) 0.69
Globulin (g/L)			1.06 (1.01-1.13) 0.044		1.06 (0.97-1.16) 0.237
CYFRA211 (ng/ml)			1.02 (1.01-1.04) 0.02		1.02 (0.99-1.04) 0.177
BMI			0.87 (0.77-0.98)		0.84 (0.7-1.01)
(kg/m2)			0.025		0.061

irAEs, immune-related adverse events; ECOG, Eastern Cooperative Oncology Group; PD-L1, programmed death ligand 1; CYFRA21-1, serum keratin19 fragment; BMI, Body mass index. P-values <0.05 were considered statistically significant.

### Impact of irAEs on prognosis

**Figure 2:**
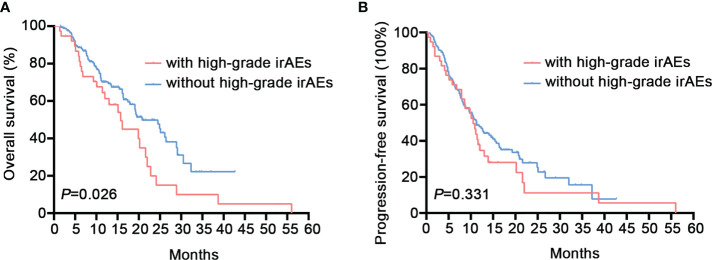
Overall Survival (OS) and progression-free survival (PFS) of patients with high-grade immune-related adverse events (irAEs). **(A)** The median OS was 15.7 (95% CI: 11.6-19.8) months for patients with high-grade irAEs vs. 20.8 (95% CI: 16.0-25.5) months for patients without high-grade irAEs (P=0.026). **(B)** The median PFS was 10.5 (95%CI: 8.8-12.3) months for patients with high-grade irAEs vs. 11.3 (95%CI: 9.0-13.6) months for patients without high-grade irAEs (P=0.331).

Our research was followed up for an average of 13.8 months, with a maximum follow-up of 56.0 months. The longest interval before the commencement of irAEs was 23.2 months, while the median was 2.8 months. More than half (52.5%) of patients had their first irAEs within 3 months of dosing. The median PFS and OS for patients in the irAEs group were 11.1 (95% CI: 8.5-13.6) and 19.5 (95% CI: 14.6-24.4) months, respectively. In the non-irAEs group, the median PFS was 11.1 (95%CI: 9.5-12.7) months (P=0.283) (**Supplementary Figure S2A**), and the OS was 20.8 (95%CI: 14.3-27.2) months (P=0.986) (**Supplementary Figure S2B**). Neither PFS nor OS differed significantly between the groups. However, a lower median OS was found for patients suffering high-grade irAEs compared to those without (15.7 vs. 20.8 months, 95% CI: 11.6-19.8 vs. 16.0-25.5, P =0.026) (**Figure 2A**). No discernible difference was found in median PFS (10.5 vs. 11.3 months, 95% CI: 8.8-12.3 vs. 9.0-13.6, P=0.331) (**Figure 2B**). When we divided patients into multiple (more than two types) irAEs group and non-multiple irAEs group, both PFS and OS were not statistically different (**Supplementary Figures S2C, D**). Additionally, when we used the median as a threshold to separate high and low AEC groups, there were no discernible changes in PFS or OS. (**Supplementary Figures S2E, F**). When we grouped according to the organ with the most frequent irAEs (skin, lung, thyroid, liver), no differences in PFS and OS were found across groups (**Supplementary Figures S3A–H**).

## Discussion

Immunotherapy has revolutionized the therapeutic strategy for advanced NSCLC. PD-1/PD-L1 antibody inhibitors exert antitumor effects by obstructing the interaction between PD-L1 and its receptor PD-1 (12, 13). With the excessive activation of cellular immunity, adverse events also occur (14, 15). With the widespread clinical use of ICIs, irAEs have also received great attention. More and more oncologists are focusing on irAEs. Multiple studies have demonstrated that irAEs are considered markers of clinical benefit from immunotherapy (5, 16–18). Some scholars have also proposed that irAEs can be predicted by peripheral blood indicators (11, 19, 20). Based on a retrospective analysis of 3164 patients in NSCLC, Cathcart-Rake et al. found that the cumulative risk of irAEs increased over time (21).

IrAEs are currently graded by CTCAE 5.0. In our study, grade 1-2 AEs occurred at a high rate (57.3%), but toxicity was mild with limited impact on patients. Grades 3-5 tended to cause a high discontinuation rate and were at risk of death. It has been reported that high-grade AEs are more prevalent in real life than in clinical trials (22, 23). Our research confirmed this with a high rate of serious AEs of 17.8%. However, few scholars have conducted in-depth research specifically on high-grade AEs. The current literature focuses on pan-cancer and overall adverse events. In a study of solid tumors, high serum albumin levels were discovered to be a risk factor for irAEs (24). Another retrospective analysis in the field of pan-cancer found that patients with high BMI had a higher probability of experiencing irAEs (25). Patients who experienced severe immune-related pneumonitis had a significant morbidity and mortality rate, according to a retrospective investigation by Suresh et al. in NSCLC, but no additional research into predictors was done (26). Recent research in solid tumors by Ruste et al. revealed that lung cancer and poor performance status were separate risk factors for severe irAEs (27). Younger age was linked to more frequent severe irAEs and more hospitalizations, according to another retrospective research in melanoma (28). We think that high-grade adverse events are of greater significance in clinical practice in NSCLC, and it is necessary to explore predictors to help early identification of serious adverse events. Our research fills this gap.

In our NSCLC cohort of 213 individuals, the most common irAEs (grades 1-5) occurred in skin (20.2%), lung (11.7%), thyroid (11.3%), and liver (10.3%). The results changed when we focused on high-grade (grade 3-5) AEs. A total of 38 patients (17.8%) had high-grade adverse events, with the main target organs being lung (8.5%), liver (3.8%), heart (1.9%), and skin (1.4%). The findings of Huang et al. in NSCLC are basically consistent with ours (29). Immune-related pneumonitis accounted for the highest proportion of grade 3-5 AEs, and elevated transaminases ranked second. Also, we observed that the retrospective study by Molina et al. suggested that irAEs requiring hospitalization occurred mainly in the gastrointestinal tract, lung, and liver (30), probably because their patient cohort included various tumor types receiving immunotherapy.

We explored risk factors for high-grade AEs. In the real world, NSCLC patients with an ECOG PS of 2-4 represent a large proportion but are underrepresented in clinical trials. Large clinical trials including Keynote-042, Keynote-010, and Keynote-024 all excluded patients with ECOG PS of 2-4 (31). In this cohort, up to 38.5% of patients had a baseline ECOG score of 2-4. The univariate analysis forest plot showed that ECOG PS of 2-4 had a strong correlation with grade 3-5 irAEs (HR 3.02, 95%CI: 1.46-6.21, P=0.003). A retrospective study from Ksienski et al. came to similar conclusions to ours, that patients with ECOG PS 2/3 were more likely to experience grade 3-5 irAEs (32). Our univariate logistic analysis also showed that BMI was negatively associated with high-grade irAEs (HR 0.87, 95% CI: 0.77-0.98, P=0.025). Previous studies have focused more on the relationship between BMI and prognosis (33, 34), and there is relatively little research on BMI and adverse events. A retrospective study in pan-cancer showed a positive association between obesity and irAEs (35). This is inconsistent with our findings in NSCLC. According to the results of our multivariate analysis, the evidence of high ECOG and low BMI as independent risk factors is not sufficient, and further prospective researches are needed in the future. Multivariate logistic regression revealed that AEC (HR 6.58, 95% CI: 1.5-28.8, P=0.012) was an independent predictor of high-grade AEs. We analyzed the relationship between organ-specific high-grade irAEs and AEC separately and found that only immune-related pneumonitis was associated with AEC (HR = 5.98, 95% CI: 1.24-28.8, P = 0.026) (**Supplementary Table S1**), which agrees with the conclusions reached by Chu et al. (36).

The precise mechanism of irAEs is currently unknown. It is currently believed that while reactivating the anti-tumor function of T cells, ICIs disrupt immune balance and cause inflammatory responses (37). Eosinophils have been extensively studied as key cells in cellular immunity. A retrospective study in colorectal cancer revealed that a higher baseline AEC was associated with better outcomes (38). The research from Nakamura et al. in melanoma showed that the occurrence of endocrine irAEs was positively linked to baseline eosinophil counts (39). Multiple studies analyzing tissue biopsy samples from patients with hepatic irAEs have found massive infiltration of inflammatory cells, primarily CD8+ T lymphocytes and eosinophils (40–43). Similarly, an autopsy analysis of a melanoma patient with multiple irAEs showed a marked increase of CD8+ T lymphocytes in involved organs such as the heart, brain, and lungs (6, 44). In addition, Naidoo et al. found infiltration of eosinophils in tissue biopsies from patients with suspected immune-related pneumonitis (6). It is well recognized that eosinophils influence the function and recruitment of lymphocytes (45). In a mouse model, Carretero et al. found that eosinophils produce chemokines like CCL5, CXCL9, and CXCL10, which recruit CD8+ T cells (46). We speculate that eosinophils promote the occurrence of irAEs by increasing the infiltration of activated CD8+ T cells. It is still necessary to investigate the specific mechanism in more detail. Due to the limitations of retrospective studies, we were unable to obtain patients’ tissues for further research. However, we make reference to the fact that Reschke et al. used flow cytometry to examine peripheral blood samples from melanoma patients receiving ICIs and discovered that PD-1 downregulation had the greatest impact on CD3+, CD4+, and CD8+ T cells at all time points after treatment initiation and that CD8+CD38+ T cells and CD8+ effector memory T cells were higher at the time point of AEs (47). Kotwal et al. compared 10 patients with ICIs-induced thyroiditis with healthy thyroid samples (n=5) by flow cytometry of thyroid fine needle aspirates and found a significant increase of CD8+ T lymphocytes in the thyroid gland of patients with ICIs-induced thyroiditis (48). Additionally, Yasuda et al. established a mouse model of thyroid-irAE induced by PD-1-Ab injection and found that the development of thyroiditis could be prevented by prior CD8+ T cell and CD4+ T cell depletion (49). Therefore, activated CD8+ T cells may predict adverse tumor responses.

Prognostic studies on patients with irAEs have consistently presented conflicting results. Multiple retrospective investigations demonstrate that immunotherapy is more effective for individuals who experience irAEs (7, 18, 50, 51). In our study, overall prognosis did not differ significantly between patients with and without irAEs. However, we noticed a shorter median OS in individuals with high-grade irAEs (15.7 vs. 20.8 months, P=0.026). Similarly, a meta-analysis in pan-cancer showed that grade 3-4 irAEs tended to lead to worse OS (52). Additionally, the research by Ksienski et al. in NSCLC revealed that individuals with a worse ECOG score had a shorter OS (24). A pan-cancer study by Paderi et al. showed that systemic steroid therapy for severe irAEs often predicted poor prognosis (53). This may help explain our conclusions. At present, most clinical studies exclude patients with an ECOG score of 2-4 and those with serious underlying diseases. However, most of the cancer patients are the elderly, and there are not a few patients with serious underlying diseases, which may cause a considerable part of the population to be ignored. In contrast, we describe the clinicopathological traits and prognosis of NSCLC individuals experiencing immunotherapy in real life. We did not have positive results for survival analysis of organ-specific adverse events, indicating that grade rather than organ type is a more worthwhile study topic for patients with adverse events.

There are a few restrictions on this study. First, the thoroughness and correctness of the data are constrained by retrospective research carried out at a single institution. Second, despite the fact that irAEs are defined in accordance with guidelines for the management of immunotherapy-related toxicity, subjectivity is inevitable while diagnosing irAEs in clinical practice. However, to our knowledge, this is one of the few studies in China that comprehensively investigates the occurrence of irAEs in real-world NSCLC patients. It is also the first study to explore predictors of serious adverse events. Interestingly, predictive techniques for irAEs may find greater relevance when immunotherapy is applied more frequently to different illness types. For instance, the anti-CD20 monoclonal antibody rituximab works by removing CD20+ B cells from the body (54). In 1997, it was first authorized for the management of CD20+ B-cell non-Hodgkin lymphoma (NHL), and ten years later, for the management of rheumatoid arthritis (RA) (55). Furthermore, rituximab is advised for broad usage in membranous nephropathy according to the most recent Kidney Disease Improved Outcome (KDIGO) guidelines (56, 57). Knowing how to anticipate the emergence of irAEs in a population of “non-oncology patients” is crucial given the immunotherapy’s growing promise. The majority of recent research on irAEs has focused on oncology patients, hence there is a need to keep exploring measures that can predict irAEs in the group of “non-oncology patients”.

In conclusion, according to our study, more than half of NSCLC patients who received immunotherapy developed irAEs. Baseline AEC was positively associated with high-grade (grade 3-5) adverse events. The survival analysis revealed that patients with serious irAEs had a worse OS. These findings broaden our knowledge of irAEs. Future prospective research is required to validate our findings.

## Data availability statement

The raw data supporting the conclusions of this article will be made available by the authors, without undue reservation.

## Ethics statement

The First Affiliated Hospital of Soochow University’s Ethics Committee granted approval for this retrospective investigation (No. 297, 2022). The patients/participants provided their written informed consent to participate in this study. Written informed consent was obtained from the individual(s) for the publication of any potentially identifiable images or data included in this article.

## Author contributions

YW, MS, and KC contributed to the conception and design of the study, the analysis of the data, and the writing of the manuscript. DL and MW participated in the acquisition of the data. YY contributed to the analysis of the data. All authors contributed to the article and approved the submitted version.

## Funding

This study was supported by National Natural Science Foundation of China. Grant Number: 81874454.

## Conflict of interest

The authors declare that the research was conducted in the absence of any commercial or financial relationships that could be construed as a potential conflict of interest.

## Publisher’s note

All claims expressed in this article are solely those of the authors and do not necessarily represent those of their affiliated organizations, or those of the publisher, the editors and the reviewers. Any product that may be evaluated in this article, or claim that may be made by its manufacturer, is not guaranteed or endorsed by the publisher.
