# Supplementary material for: Peripheral absolute eosinophil count identifies the risk of serious immune-related adverse events in non-small cell lung cancer
Source: Front Oncol. 2022 Oct 13;12:1004663. doi: 10.3389/fonc.2022.1004663 (PMC9608122; doi:10.3389/fonc.2022.1004663)
Supplement: Supplementary file 1 [file DataSheet_1.docx]

<supplementary-material xlink:href="DataSheet_1.docx" id="SM1" mimetype="application/vnd.openxmlformats-officedocument.wordprocessingml.document"/>Supplementary Material

# Supplementary Figures and Tables

## Supplementary Figures


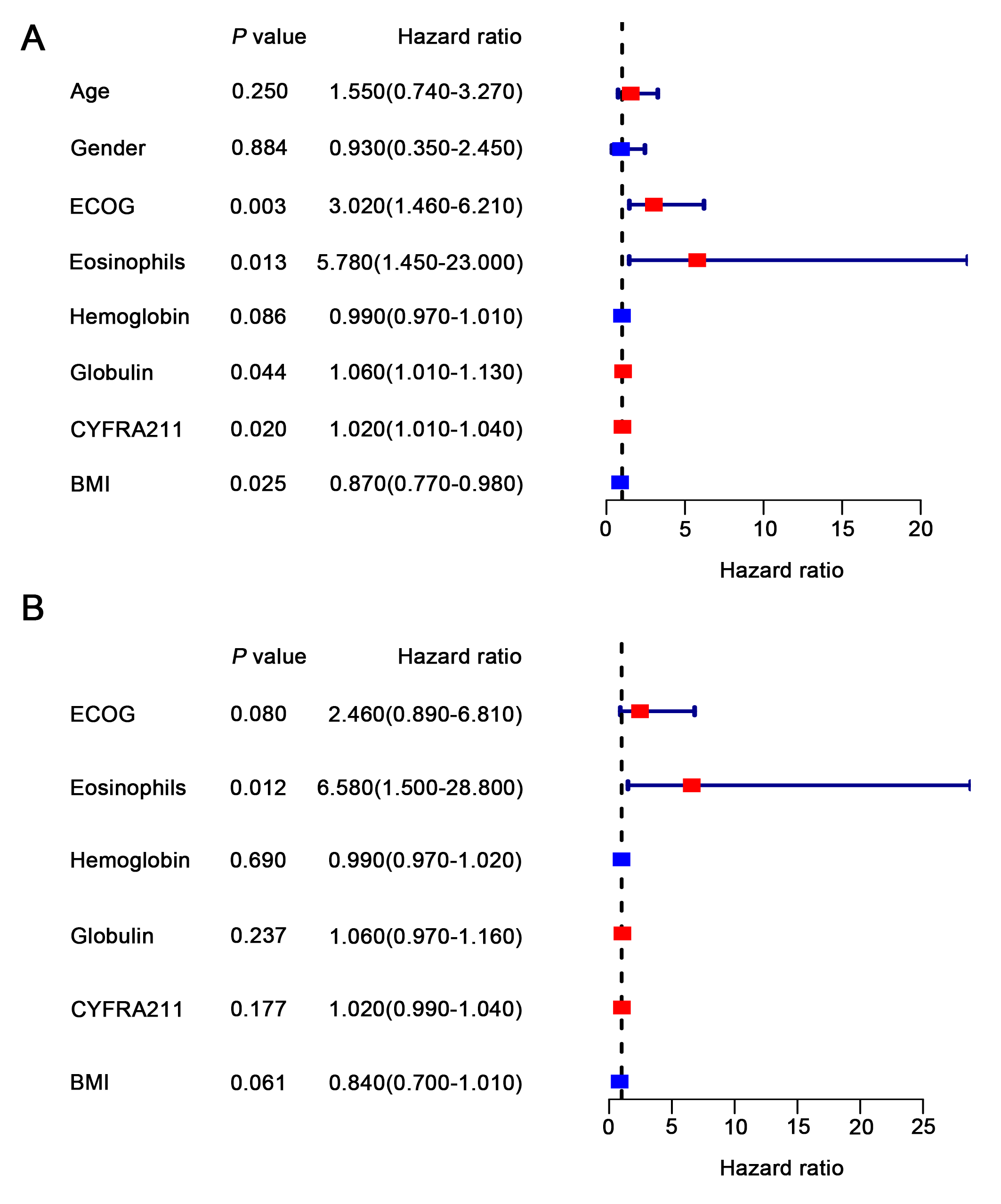


**Supplementary Figure S1.** Logistic regression with univariate (A) and multivariate (B) analyses for the risk factors of grade 3-5 irAEs. Characteristics in univariate models with p-value <0.1 were included in multivariate analysis. irAEs, immune-related adverse events; ECOG, Eastern Cooperative Oncology Group; CYFRA21-1, serum keratin19 fragment; BMI, Body mass index.

##
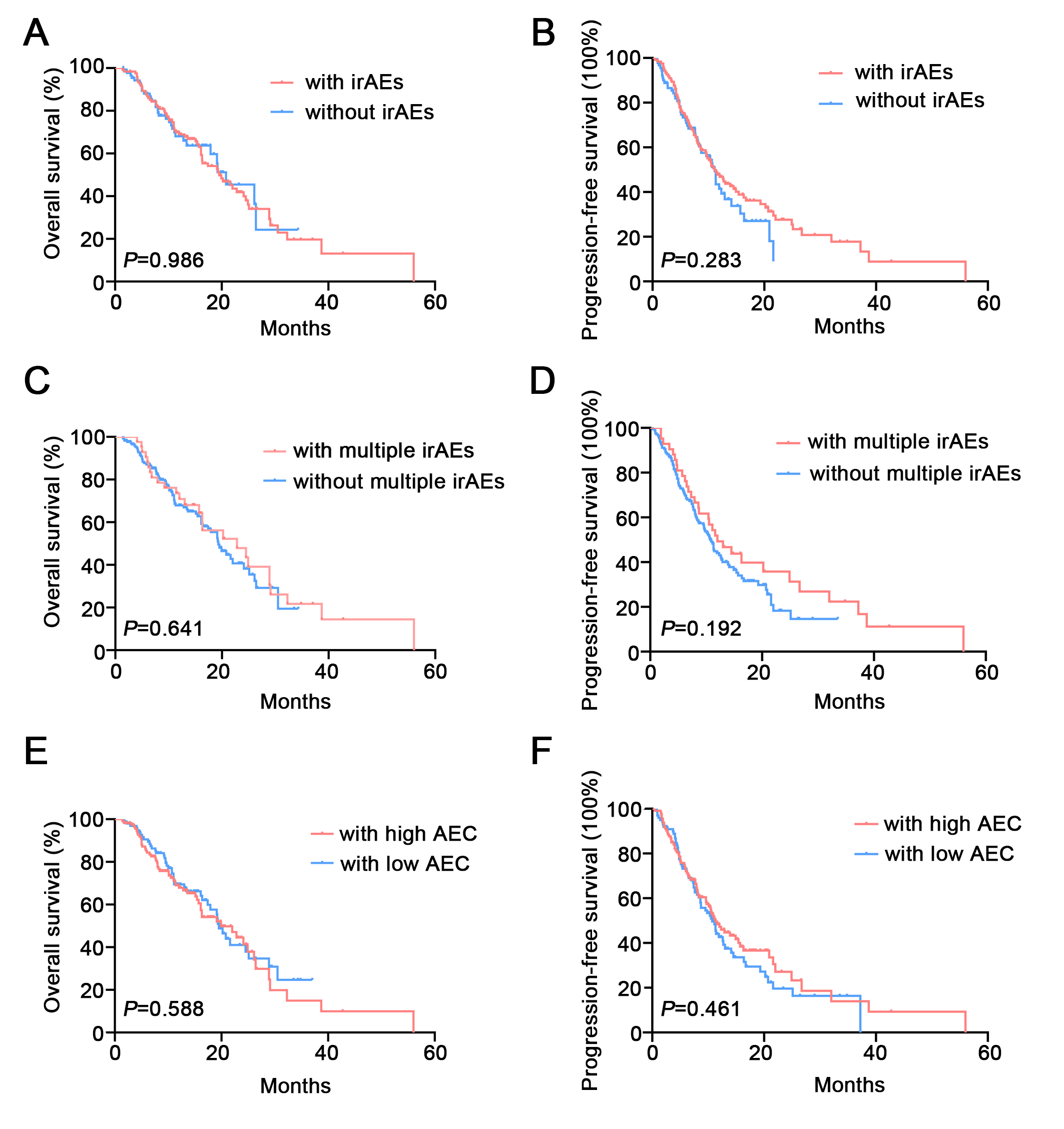


**Supplementary Figure S2.** Comparisons of overall survival (OS) and progression-free survival (PFS) in different groups. (A) The median OS was 19.5 (95% CI: 14.6-24.4) months in the irAEs group and 20.8 (95% CI: 14.3-27.2) months in the non-irAEs group (P=0.986). (B) The median PFS was 11.1 (95%CI: 8.5-13.6) months in the irAEs group and 11.1 (95%CI: 9.5-12.7) months in the non-irAEs group (P=0.283). (C) The median OS was 22.8 (95% CI: 11.2-34.4) months in the multiple irAEs group and 19.3 (95% CI: 16.7-21.9) months in the non-multiple irAEs group (P=0.641). (D) The median PFS was 12.0 (95%CI: 7.3-16.7) months in the multiple irAEs group and 10.7 (95%CI: 9.4-12.0) months in the non-multile irAEs group (P=0.192). (E) The median OS was 19.9 (95% CI: 13.1-26.6) months in the high AEC group and 19.5 (95% CI: 17.7-21.4) months in the low AEC group (P=0.588). (F) The median PFS was 11.3 (95%CI: 9.0-13.7) months in the high AEC group and 10.7 (95%CI: 8.3-13.1) months in the low AEC group (P=0.461).


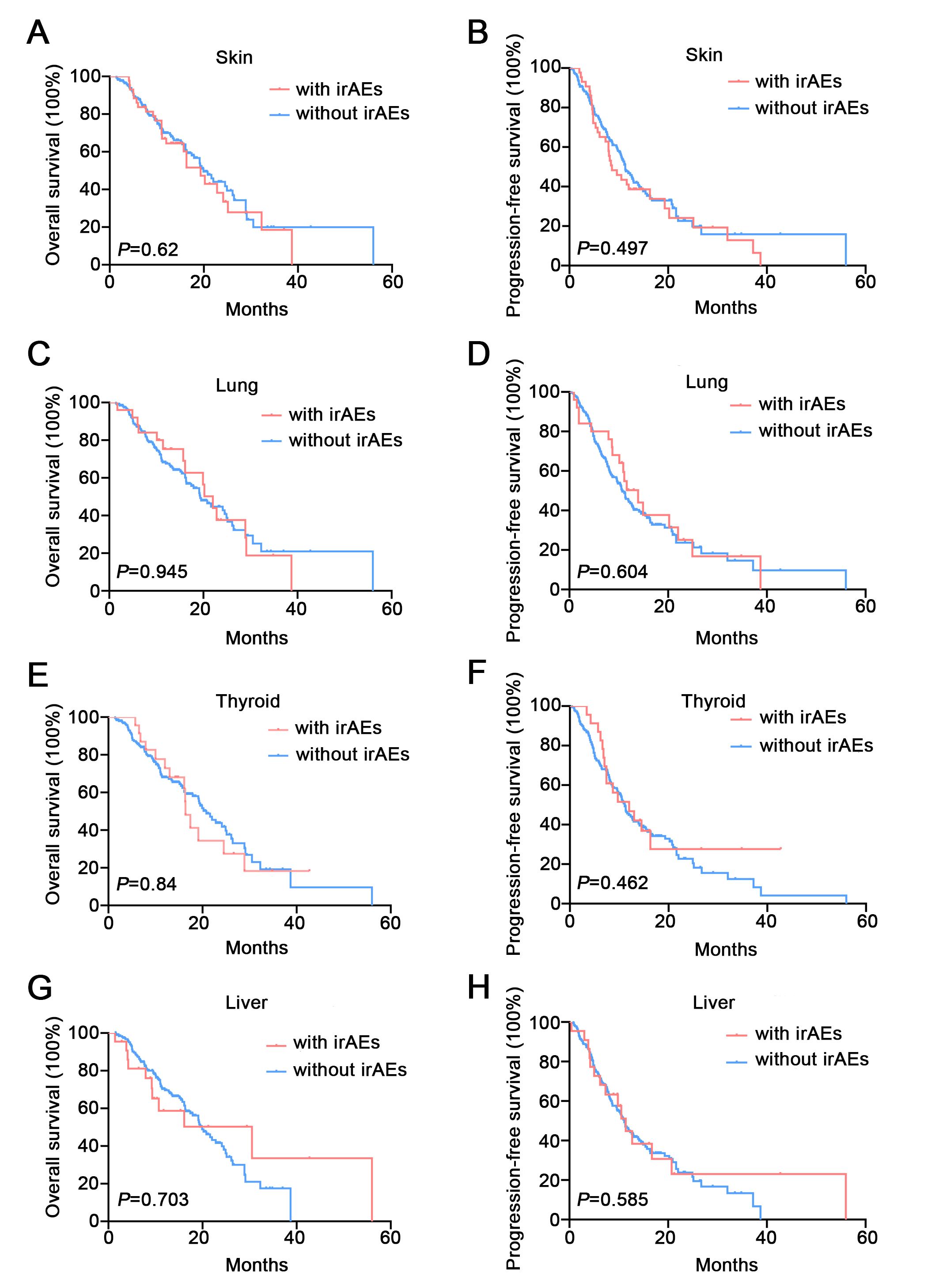


**Supplementary Figure S3.** Comparisons of overall survival (OS) and progression-free survival (PFS) in irAEs of different organs. (A) The median OS was 19.3 (95% CI: 13.8-24.8) months in the skin-irAEs group and 19.9 (95% CI: 16.7-23.1) months in the non-skin irAEs group (P=0.62). (B) The median PFS was 8.6 (95%CI: 5.5-11.6) months in the skin-irAEs group and 11.3 (95%CI: 9.7-12.9) months in the non-skin irAEs group (P=0.497). (C) The median OS was 22.0 (95% CI: 18.0-26.0) months in the lung-irAEs group and 19.3 (95% CI: 15.8-22.8) months in the non-lung irAEs group (P=0.945). (D) The median PFS was 13.9 (95%CI: 9.8-18.1) months in the lung-irAEs group and 10.6 (95%CI: 8.9-12.3) months in the non-lung irAEs group (P=0.604). (E) The median OS was 16.4 (95% CI: 14.8-17.9) months in the thyroid-group and 20.8 (95% CI: 17.9-23.6) months in the non-thyroid irAEs group (P=0.84). (F) The median PFS was 12.0 (95%CI: 5.7-18.3) months in the thyroid-irAEs group and 11.1 (95%CI: 9.8-12.3) months in the non-thyroid irAEs group (P=0.462). (G) The median OS was 16.1 (95% CI: 0.0-35.6) months in the liver-irAEs group and 19.9 (95% CI: 16.9-22.8) months in the non-liver irAEs group (P=0.703). (H) The median PFS was 11.4 (95%CI: 8.5-14.3) months in the liver-irAEs group and 11.1 (95%CI: 9.4-12.7) months in the non-liver irAEs group (P=0.585).

**1.2 Supplementary Tables**

**Supplementary Table S1.** Univariate and multivariate analyses of factors associated with severe pneumonitis.

| Factors |  |  | Univariate analysis |  | Multivariate analysis |
| --- | --- | --- | --- | --- | --- |
|  |  |  | OR(95%CI) |  | OR(95%CI) |
|  |  |  | *P* |  | *P* |
| Age | <65  ≥65  Male  Female  0-1  ≥2 | | 0.82(0.31-2.16)  0.682 |  |  |
| Gender |  |  | 3.21(0.41-25.04)  0.032 |  |  |
| ECOG |  |  | 3.57(1.28-9.93)  0.048 |  | 2.79 (0.96-8.15)  0.061 |
| Eosinophils (×10^9^/L) |  |  | 6.87(1.51-31.32)  0.013 |  | 5.98(1.24-28.8)  0.026 |
| Hemoglobin  (g/L) |  |  | 1.01(0.97-1.02)  0.669 |  |  |
| Globulin  (g/L) |  |  | 1.07(0.97-1.18)  0.19 |  |  |
| CRP  (mg/L) |  |  | 1.02(1.01-1.04)  0.097 |  | 1.02(1.01-1.04)  0.126 |
| BMI |  |  | 0.97(0.83-1.12) |  |  |
| (kg/m2) |  |  | 0.639 |  |  |
| ECOG, Eastern Cooperative Oncology Group; PD-L1, programmed death ligand 1; CRP, C-reactive protein; BMI, Body mass index. | | | | | |
